# Bevacizumab Eye Drops Vs. Intra-meibomian Gland Injection of Bevacizumab for Meibomian Gland Dysfunction-Associated Posterior Blepharitis

**DOI:** 10.3389/fmed.2022.895418

**Published:** 2022-06-10

**Authors:** Chitchanok Tantipat, Ngamjit Kasetsuwan, Patraramon Chotikkakamthorn, Krit Pongpirul

**Affiliations:** ^1^Department of Ophthalmology, Faculty of Medicine, Chulalongkorn University and King Chulalongkorn Memorial Hospital, Bangkok, Thailand; ^2^Excellence Center of Cornea and Limbal Stem Cell Transplantation, Department of Ophthalmology, King Chulalongkorn Memorial Hospital and Faculty of Medicine, Chulalongkorn University, Bangkok, Thailand; ^3^Center of Excellence for Cornea and Stem Cell Transplantation, Faculty of Medicine, Chulalongkorn University and King Chulalongkorn Memorial Hospital, Thai Red Cross Society, Bangkok, Thailand; ^4^Department of Preventive and Social Medicine, Faculty of Medicine, Chulalongkorn University, Bangkok, Thailand

**Keywords:** bevacizumab, lid hygiene, lid margin telangiectasia, meibomian gland dysfunction (MGD), vascular endothelial growth factor (VEGF)

## Abstract

**Aims:**

This study aimed to evaluate the efficacy and safety of bevacizumab eye drops compared with those of an intra-meibomian gland (MG) injection of bevacizumab when performed in conjunction with standard lid hygiene in patients with meibomian gland dysfunction (MGD)-associated posterior blepharitis.

**Methods:**

This prospective, open-label, observer-blinded randomized controlled trial included 60 eyes of 30 patients with MGD-associated posterior blepharitis who exhibited lid margin telangiectasia, treated at the Chula Refractive Surgery Center of King Chulalongkorn Memorial Hospital. Patients were randomized to receive lid hygiene plus 0.05% bevacizumab eye drops or a single intra-MG injection of 2.5% bevacizumab. All patients were instructed to perform routine lid hygiene care as demonstrated in an instructional video. Primary outcomes included telangiectasia grading and the lid margin neovascularized area (LMNA). Secondary outcomes included the Ocular Surface Disease Index (OSDI) score, corneal staining, meibum quality, meiboscore, conjunctival redness, fluorescein break-up time (FBUT), lipid layer thickness, treatment compliance, and adverse events. All parameters were evaluated before and 3 months after treatment.

**Results:**

After treatment, there were no significant differences in telangiectasia grade and LMNA between groups (mean difference, −0.14, 95% CI −0.42 to 0.15, *p* = 0.338, −0.1, 95% CI −1.1 to 0.8, *p* = 0.761, respectively); however, the injection group exhibited significant improvements in both telangiectasia grade and LMNA, while, in the eye drop group, only telangiectasia grade showed a significant improvement relative to baseline. The injection group also exhibited significant improvements in corneal staining (mean difference, −0.78, 95% CI −1.29 to −0.27, *p* = 0.003), meiboscores (mean difference, −0.37, 95% CI −0.52 to −0.21, *p* <0.001), and FBUT (mean difference, 1.25, 95% CI 0.21–2.29, *p* = 0.019) compared to the eye drop group. OSDI scores, corneal staining, meibum quality, meiboscores, and conjunctival redness significantly improved relative to baseline in both groups. No local and systemic adverse event was observed at month 3 in both groups.

**Conclusion:**

When performed with regular lid hygiene, intra-MG injection and topical application of bevacizumab are safe and effective for improving lid margin telangiectasia and the signs and symptoms of MGD-associated posterior blepharitis. This therapy may represent an alternative or adjunctive treatment for patients with MGD-associated posterior blepharitis.

## Introduction

Meibomian gland dysfunction (MGD) (1) is the leading cause of dry eye disease (DED) worldwide. MGD characteristics include chronic abnormalities of the meibomian glands (MG) and alterations in the quality of gland secretion, resulting in tear film instability. Clinical signs of MGD can occur on the posterior lid margin and include lid margin irregularities, prominent telangiectatic blood vessels, hyperplasia/metaplasia, and pouting of the MG orifices.

Telangiectasia or lid margin vascularity is a clinical sign that usually co-exists with MGD (2). Lid margin telangiectasia is one of the major signs for MGD-associated posterior blepharitis diagnosis (3). Similarly, the rise in the vascularization of the posterior lid margin indicates the increase in inflammation. Therefore, MGD with lid margin telangiectasia can be considered as MGD-associated posterior blepharitis (4, 5).

Currently, the effective conventional MGD-associated posterior blepharitis treatment is warm compresses and lid hygiene (6). Since most patients do not regularly follow the treatment process (7), outcomes can differ from the expected treatment results. Moreover, standard warm compress and lid hygiene treatment may fail to reduce lid margin telangiectasia (8). Although several medications (e.g., topical steroids) are also effective in MGD-associated posterior blepharitis treatment, they have been associated with side effects, such as ocular hypertension and cataracts (6).

Bevacizumab [an anti-vascular endothelial growth factor (VEGF)-A recombinant humanized monoclonal antibody] has been widely used in the treatment of systemic and ocular diseases (9, 10). Moreover, previous studies have reported that an intra-MG bevacizumab injection (11) can decrease lid margin telangiectasia by up to 42%, in addition to improving dry eye symptoms. However, to our best knowledge, no randomized controlled trials have investigated the use of bevacizumab eye drops in patients with MGD-associated posterior blepharitis compared with the use of intra-MG injections.

In this study, we aimed to investigate whether treatment with topical or intra-MG bevacizumab in conjunction with standard lid hygiene can help to reduce lid margin telangiectasia and improve the signs and symptoms of MGD-associated posterior blepharitis.

## Materials and Methods

This study was conducted at the Chula Refractive Surgery Center of King Chulalongkorn Memorial Hospital (Bangkok, Thailand) from September 2020 to May 2021, approved by the hospital's Institutional Review Board (IRB Certificate of Approval No. 947/2020), and followed the tenets of the Declaration of Helsinki. The Thai Clinical Trial Registry number was TCTR20201102001.

### Patients

The study included patients with MGD-associated posterior blepharitis attending the Ophthalmic Outpatient Department. The inclusion criteria were age 18–80 years and ≥1 of the following symptoms: dryness, foreign body sensation, burning, tearing, and duration of >6 months; diagnosis of MGD stage 2 or 3 (6) with lid margin telangiectasia grade 2 or 3 (12) in both eyes; and willingness to undergo regular follow-up appointments. The exclusion criteria were structural ocular abnormality; history of ocular trauma; history of ocular/other surgeries; use of any treatment for DED or MGD, except artificial tears, within the past month; active allergy, infection, or inflammation at the ocular surface unrelated to DED or MGD; history of ocular herpes infection (13); lacrimal gland drainage system abnormality; contact lens wear within the past month; use of systemic medication affecting the ocular surface, systemic anti-inflammatory medication, anticoagulants, or antiplatelet medication; unstable systemic diseases, such as uncontrolled hypertension, uncontrolled diabetes mellitus, stroke, coronary artery disease, cerebrovascular disease, and bleeding diathesis; history of bevacizumab contraindications, including congestive heart failure, gastrointestinal perforation, pregnancy, breast feeding, reversible posterior leukoencephalopathy syndrome, proteinuria, or surgical/wound healing complications; and allergy to bevacizumab or moxifloxacin.

To determine the adequate sample size, power calculations were made according to neovascularized area reduction as the designated outcome. The percentage reduction of corneal neovascularization as a result of bevacizumab eye drop was 29% (10) and the percentage reduction of eyelid neovascularization as a result of an intra-MG bevacizumab injection was 42% (11). With a *p*-value of <0.05 and a study power of 90%, we determined a sample size of 13 patients per group. The standard deviation of normal values was equally estimated to be 10%. After adjusting for a 20% dropout rate, 15 patients were required in each group to detect a significant difference of 13% in pairwise comparisons. The sample size calculation was determined using Stata Statistical Software (StataCorp LLC; College Station, TX, USA).

### Experimental Design

**Figure 1:**
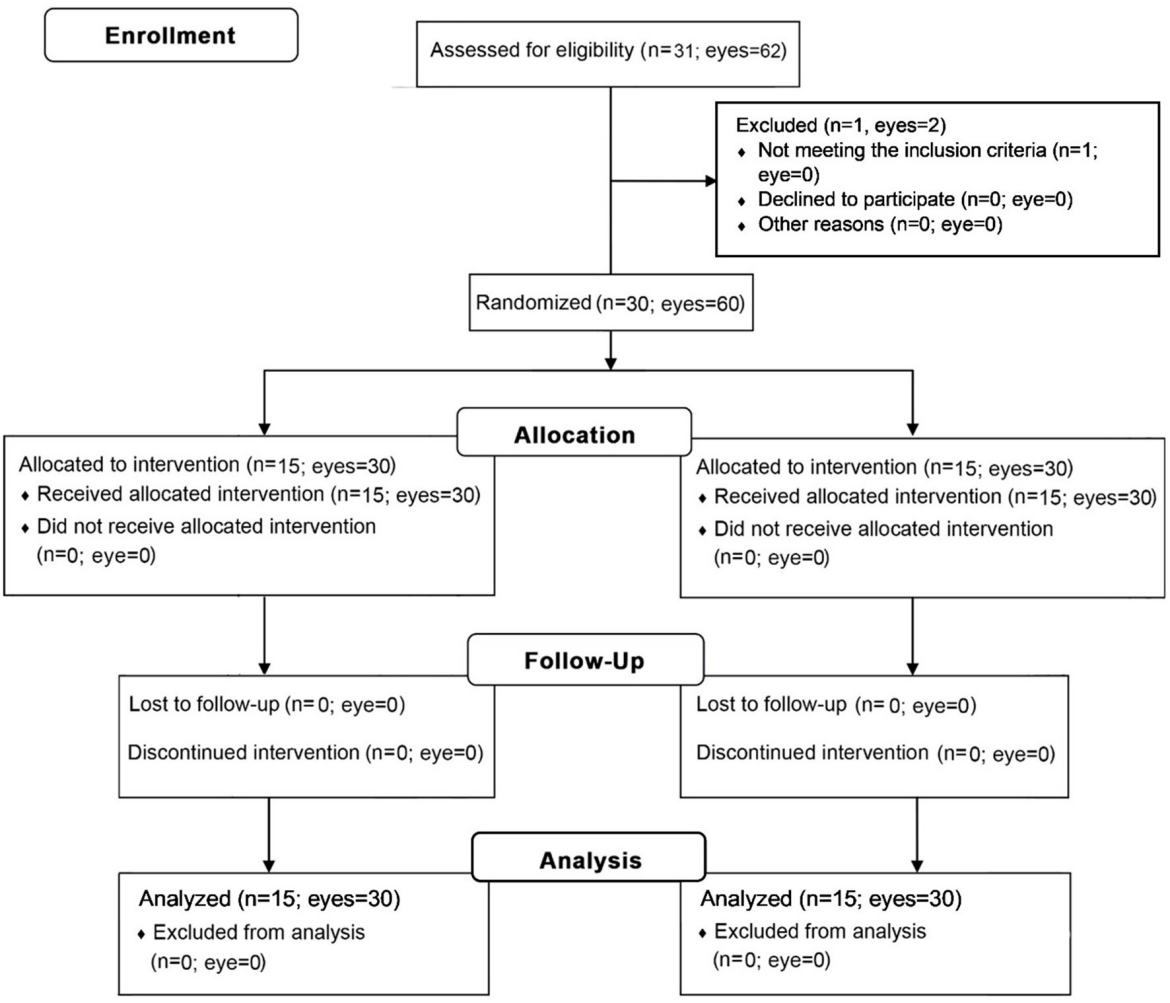
Consolidated standards of reporting trials flow diagram.

After obtaining informed consent and collecting baseline data, we divided the patients into an intra-MG bevacizumab injection group and a bevacizumab eye drop group *via* computer-generated randomization with a block size of 4. Group assignments were concealed from the investigator by an independent third party (Figure 1). All patients were instructed to perform a video-demonstrated lid hygiene care. During the first visit, another surgeon (N.K.) administered bevacizumab injections into both eyes of each patient in the injection group. On the following day, the same surgeon evaluated the patients for postoperative complications. Bevacizumab eye drops were then administered to the eye drop group, who were instructed to apply them to both eyes four times a day. During this period, all participants were allowed to use previous ocular lubricants as needed. After the first visit, re-examinations were performed at 1 week and at 1, 2, and 3 months. All clinical measurements were performed in both eyes by a single-blinded investigator.

### Outcome Assessment

**Figure 2:**
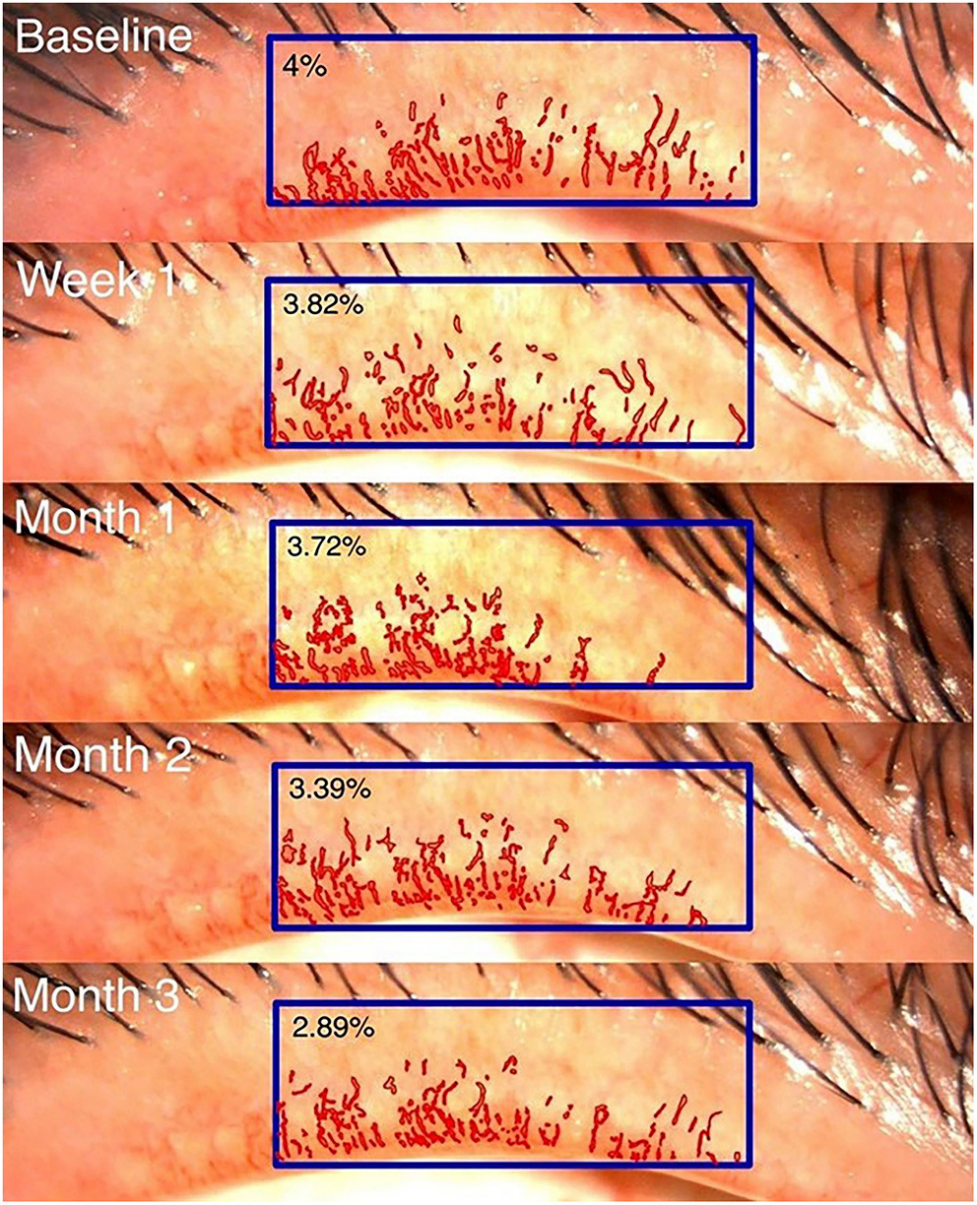
Image of the lid margin neovascularized area.

The primary outcomes were telangiectasia grade (12) ranging from 0 (no telangiectasia) to 3 (severe telangiectasia) and computer-assisted quantitative measurements of lid margin telangiectasia. The latter were obtained by repeatedly composing standardized digital slit-lamp images, which were then analyzed morphometrically using image analysis software (9) (Cell Sens Dimension software: Olympus, Hamburg, Germany) by a single outsource technician. The lid margin neovascularized area (LMNA) was measured in pixels. Its ratio to the central one-third of the lid margin area was calculated as the LMNA percentage (Figure 2). Secondary outcomes included the Ocular Surface Disease Index (OSDI) score, corneal staining, meibum quality score, meiboscore, conjunctival redness, fluorescein break-up time (FBUT), and lipid layer thickness (LLT).

Dry eye symptoms were assessed using the OSDI questionnaire. The total OSDI score ranges from 0 (no symptoms) to 100 (more severe symptoms) (14). Lid margin telangiectasia (12) was graded as previously described. Conjunctival redness was graded from 0 (no redness) to 4 (severe redness involving the sclera) using the Institute for Eye Research scale (15). FBUT was recorded as the average of three measurements obtained using 5-μL volume of 2% Na-fluorescein. Corneal staining scores were graded using the modified Oxford grading scheme (16), in which scores range from 0 (less severe) to 5 (more severe). The meibum quality score was assessed using an MG evaluator (TearScience Inc., Morrisville, NC, USA), which was pressed against a total of eight MGs in the central area of the lower lid margin. Meibum secretion was assessed and graded on a scale from 0 to 3 (0, clear liquid secretion; 1, cloudy liquid secretion; 2, cloudy particulate secretion; 3, inspissated/toothpaste consistency). A total score of 0–24 was recorded (6).

LLT was measured using a LipiView instrument (TearScience Inc., Morrisville, NC, USA). The interferometer was used to analyze LLT in interferometric color units (ICUs), where one ICU is equal to 1 nm of LLT. The meiboscore was determined using non-contact meibography (Keratograph 5M; Oculus Optikgeräte GmbH, Wetzlar, Germany). Infrared images of both the upper and lower lids were captured. The score was graded based on the percentage of atrophic MGs, ranging from 0 to 3 (0, 0%; 1, <33%; 2, 33–67%; 3, >67%). (17).

Local or systemic adverse events (AEs) were assessed the day after (only in the injection group) and at 1 week and 1, 2, and 3 months after treatment. Compliance with lid hygiene was evaluated based on the frequency at which the procedure was performed per week.

### Combined Treatment With Bevacizumab Eye Drops and Standard Lid Hygiene

#### Standard Lid Hygiene

During the first visit, all patients watched the lid hygiene video. The lid hygiene care began with application of a warm compress (42°C) for 5 min, following which an eyelid massage was performed by applying pressure toward the lid margin of both upper and lower eyelids. Subsequently, the eyelids were cleaned with water and dried with a towel. Patients were requested to perform this procedure at least twice daily.

#### Bevacizumab Eye Drops

Bevacizumab (Avastin, F. Hoffmann La-Roche AG, Switzerland) 100 mg/4 mL was prepared from its intravenous (IV) form diluted in normal saline solution (NSS) under laminar flow. The 0.05% bevacizumab was transferred into 5-mL eye dropper bottles and stored at −20°C. Participants were advised to apply the eye drops four times a day and store them at 4°C during usage.

### Combined Treatment With Intra-meibomian Gland Bevacizumab Injection and Standard Lid Hygiene

#### Intra-meibomian Gland Bevacizumab Injection

**Figure 3:**
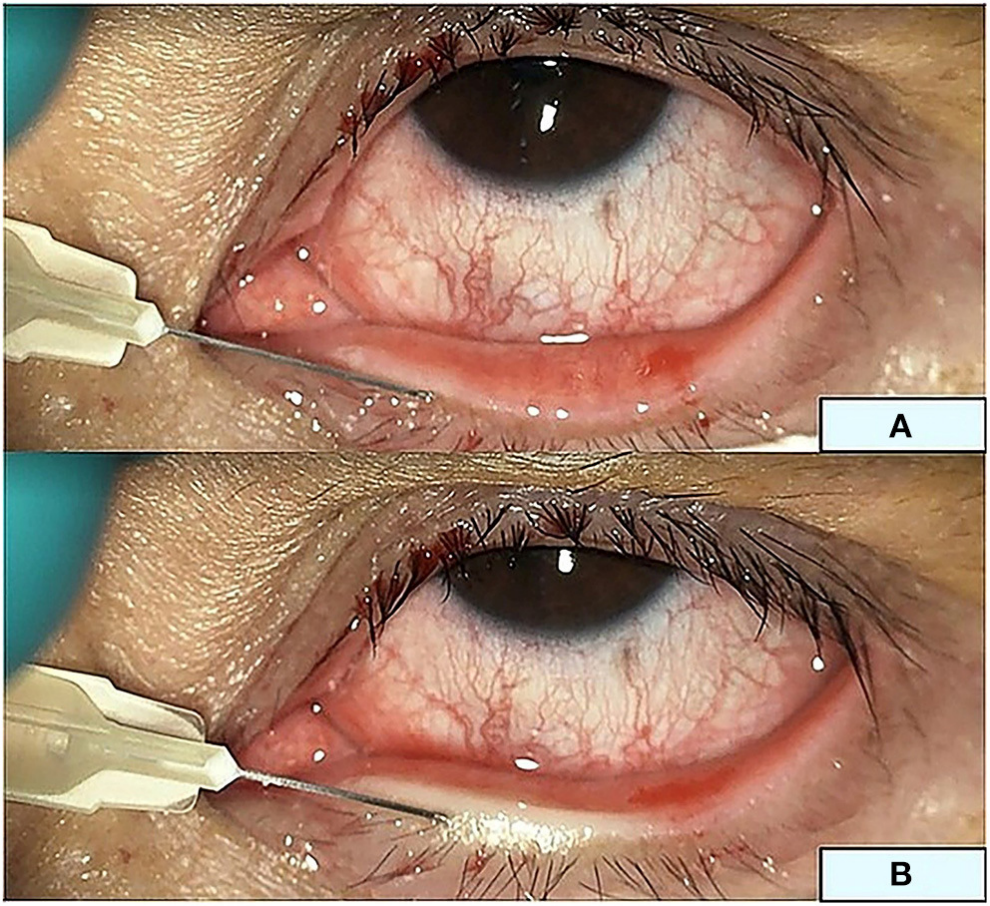
Intra-meibomian gland injection. Three sites at the upper eyelid and two sites at the lower eyelid margin **(A)** for a total injection volume of 150 μL. **(B)** The lower eyelid during injection.

For intra-MG injection, 2.5% bevacizumab was prepared from the IV form, transferred into a 1-mL syringe under laminar flow, and stored at 4°C. A single bevacizumab injection was performed in the minor operating room of the Chula Refractive Surgery Center. Subsequently, 10% povidone iodine was applied to the skin for 3 min and wiped off with NSS. Tetracaine eye drops were applied to the conjunctival sac, and 4% lidocaine gel was directly applied to the lid margin with a sterile cotton-tipped applicator. Contact lenses were placed on the cornea. Intra-MG injections were performed at a depth of 1–2 mm using a 30-gauge needle at the vascular-enriched lid margin tissue around the MG orifices. Injections were performed by an expert surgeon (N.K.) at five sites per eye (Figure 3).

### Statistical Analysis

Baseline characteristics, including sex, age, systemic comorbidities, and other factors, were assessed using descriptive statistics. A generalized estimating equation was used to analyze longitudinal data with uneven time points (i.e., OSDI score, telangiectasia grade, LMNA, corneal staining, meibum quality, meiboscore, conjunctival redness, FBUT, and LLT). Fisher's exact test was performed to compare nominal data. An independent samples *t*-test was performed to compare continuous data. The probability of improvement in the telangiectasia grade by more than 1 point was determined using the Kaplan–Meier method with log-rank testing. The correlation between telangiectasia grade and LMNA was examined using mixed model analysis. The statistically significant *p*-value was <0.05, and statistical analyses were performed using Stata Statistical Software (StataCorp LLC; College Station, TX, USA).

## Results

We enrolled 31 patients in the treatment program. One patient was excluded owing to severe MGD-associated posterior blepharitis signs and symptoms resulting in 15 patients per group. There were no differences in demographic or baseline clinical data between the groups, except for a shorter level of FBUT in the injection group (Table 1).

**Table 1:** Baseline characteristics.

**Variables**	**Injection group (*n* = 15)**	**Eye drop group (*n* = 15)**	***p*-value**
Female	11 (73.3%)	14 (93.3%)	0.33
Age (years), mean ± SD	63.8 ± 8.14	63.4 ± 6.03	0.88
**Systemic comorbidities**			
Hypertension	3 (20%)	7 (46.7%)	0.245
Dyslipidemia	2 (13.3%)	7 (46.7%)	0.109
Diabetic mellitus	0 (0%)	2 (13.3%)	0.48
Others	5 (33.3%)	6 (40%)	0.215
**Ocular comorbidities**			
Ocular trauma	0 (0%)	0 (0%)	NA
Ocular surgery	2 (13.3%)	1 (6.7%)	1
Ocular diseases	0 (0%)	1 (6.7%)	1
**Ophthalmic medications**			
Artificial tears	14 (93.3%)	14 (93.3%)	1
Lubricant eye gels	6 (40%)	5 (33.3%)	1
**Non-ophthalmic medications**			
Antihypertensive medications	2 (13.3%)	8 (53.3%)	0.05
Antihyperlipidemic medications	3 (20%)	9 (60%)	0.06
Antidepressants	3 (20%)	1 (6.7%)	0.598
Others	6 (40%)	8 (53.3%)	0.715
**Patient-reported outcome**			
OSDI score (1–100) mean ± SD	25.45 ± 14.28	23.73 ± 9.94	0.705
**Primary clinical outcomes**			
Telangiectasia grading (0–3), mean ± SD	2.23 ± 0.59	2.3 ± 0.53	0.748
Grade 1	3 (10%)	1 (3.3%)	0.438
Grade 2	15 (50%)	19 (63.3%)	
Grade 3	12 (40%)	10 (33.3%)	
Lid margin neovascularized area (%), mean ± SD	4.6 ± 2.3	5.9 ± 3.2	0.213
**Secondary clinical outcomes**			
Corneal staining (0–5), mean ± SD	1.47 ± 1.27	0.87 ± 1.09	0.177
Meibum quality (0–24), mean ± SD	19.02 ± 3.82	18.79 ± 3.74	0.545
Meiboscore (0–6), mean ± SD	2.21 ± 1.42	1.68 ± 1.11	0.261
Conjunctival redness (0–4), mean ± SD	0.77 ± 0.78	0.73 ± 0.7	0.903
FBUT (s), mean ± SD	3.64 ± 1.52	4.88 ± 1.64	0.041*
LLT (nm), mean ± SD	64 ± 26.24	72.33 ± 27.17	0.4

*OSDI, Ocular Surface Disease Index; FBUT, fluorescein break-up time; s, second; nm, nanometer; LLT, lipid layer thickness; NA, not applicable*.

### Ocular Surface Disease Index

**Figure 4:**
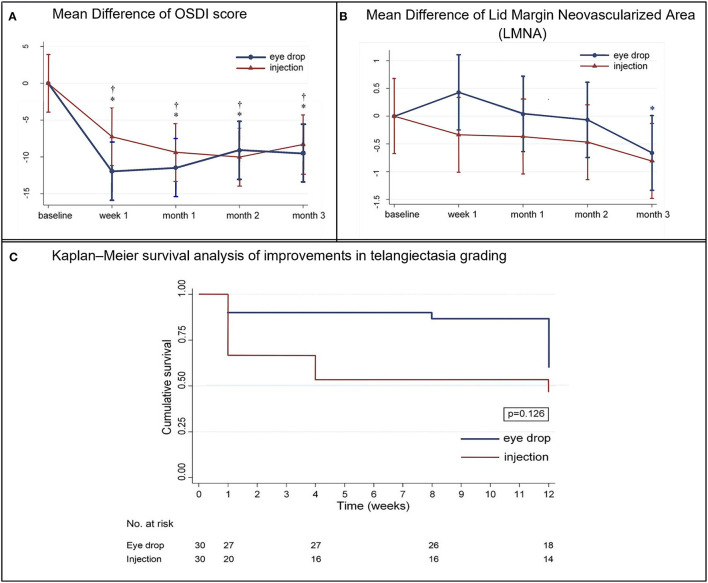
**(A)** Mean (±standard deviation) difference in the Ocular Surface Disease Index (OSDI) scores from baseline at each visit. ^†^*p* < 0.05, within-eye drop group differences. *p < 0.05, within-injection group differences. **(B)** Mean (±standard deviation) difference in lid margin neovascularized area from baseline at each visit. *p < 0.05, within-injection group differences. **(C)** Kaplan–Meier survival analysis of improvements in telangiectasia grading.

In the eye drop group, the OSDI scores significantly decreased from 23.73 to 11.73 at week 1 [mean change, −11.93, 95% confidence interval (CI), −16.15 to −7.7, *p* <0.001], persisting until month 3 (*p* = 0.234) (Supplementary Table 1; Figure 4). In the injection group, the OSDI scores significantly decreased from 25.45 to 18.18 at week 1 (mean change, −7.27, 95% CI, −11.11 to −3.42, *p* <0.001), persisting until month 3 (*p* = 0.213). There was no significant difference between the groups at 3 months (mean difference, 0.99, 95% CI −4.79 to 6.77, *p* = 0.738).

### Telangiectasia Grading and Lid Margin Neovascularized Areas

The Kaplan–Meier survival analysis (Figure 4) revealed that, in the injection group, the probability of improving the telangiectasia grade by more than 1 level was 33.3% at week 1, which increased to 53.3% at month 3 post-treatment. In the eye drop group, the probability of achieving such an improvement was 13.3% at month 2, which increased to 40% at month 3. However, there was no significant difference between the groups at 3 months (*p* = 0.126). In the injection group, the telangiectasia grade decreased from 2.23 to 2.05 at month 1 (mean change, −0.26, 95% CI −0.48 to −0.04, *p* = 0.22) and 3 (mean change, −0.56, 95% CI −0.56 to −0.33, *p* <0.001); in the eye drop group, the telangiectasia grade decreased significantly from 2.3 to 2.1 at month 2 (mean change, −0.2, 95% CI −0.37 to −0.03, *p* = 0.024) and significantly improved by month 3 (*p* = 0.015). There was no between-group difference in the telangiectasia grade at month 3 (mean difference, −0.14, 95% CI −0.42 to 0.15, *p* = 0.338) (Table 2).

**Table 2:** Primary outcomes.

**Telangiectasia**	**Injection group**			**Eye drop group**			**Between treatment**	
**grading (0–3)**	**Mean ±SD**	**Mean change (95% CI)**	***p*-value**	**Mean ±SD**	**Mean change (95% CI)**	***p*-value**	**Mean difference (95% CI)**	***p*-value**
Baseline	2.23 ± 0.59	Reference	1	2.3 ± 0.53	Reference	1	Reference	1
1 week	2.07 ± 0.68	−0.17 (−0.39, 0.06)	0.141	2.22 ± 0.66	−0.13 (−0.31, 0.04)	0.134	−0.03 (−0.31, 0.25)	0.817
1 month	2.05 ± 0.38	−0.26 (−0.48, −0.04)	0.022*	2.2 ± 0.62	−0.08 (−0.26, 0.09)	0.348	−0.18 (−0.46, 0.11)	0.223
2 months	2.17 ± 0.59	−0.08 (−0.31, 0.14)	0.461	2.1 ± 0.54	−0.2 (−0.37, −0.03)	0.024*	0.12 (−0.16, 0.4)	0.417
3 months	1.89 ± 0.86	−0.56 (−0.78, −0.33)	<0.001*	1.9 ± 0.54	−0.42 (−0.59, −0.24)	<0.001*	−0.14 (−0.42, 0.15)	0.338
**Lid margin neovascularized area (%)**	**Injection group**			**Eye drop group**			**Between treatment**	
	**Mean** **±SD**	**Mean change (95% CI)**	***p*** **-value**	**Mean** **±SD**	**Mean change (95% CI)**	***p*** **-value**	**Mean difference (95% CI)**	***p*** **-value**
Baseline	4.6 ± 2.3	Reference	1	5.9 ± 3.2	Reference	1	Reference	1
1 week	4.2 ± 2.1	−0.3 (−0.9, 0.2)	0.248	6.4 ± 4.4	0.4 (−0.3, 1.2)	0.255	−0.8 (−1.7, 0.2)	0.108
1 month	4.2 ± 2.3	−0.4 (−0.9, 0.2)	0.208	6 ± 3.6	0 (−0.7, 0.8)	0.922	−0.4 (−1.3, 0.5)	0.396
2 months	4.1 ± 2.1	−0.5 (−1, 0.1)	0.105	5.9 ± 3.5	−0.1 (−0.8, 0.7)	0.858	−0.4 (−1.3, 0.5)	0.395
3 months	3.8 ± 2	−0.8 (−1.4, −0.2)	0.005*	5.3 ± 3	−0.7 (−1.4, 0.1)	0.077	−0.1 (−1.1, 0.8)	0.761

Subjective and objective outcomes. *p-value <0.05.

Details regarding the pre- and post-treatment LMNA in both groups are presented in Table 2 and Figure 4. In the injection group, the LMNA decreased from 4.6 to 4.2% at 1 week post-treatment (mean change, −0.3%, 95% CI −0.9 to 0.2, *p* = 0.248) and remained stable until month 2. At 3 months, LMNA had significantly decreased to 3.8% (mean change, −0.8%, 95% CI −0.8 to 0.2, *p* = 0.005). In the eye drop group, LMNA decreased from 5.9 to 5.3% (mean change, −0.7%, 95% CI −1.4 to 0.1, *p* = 0.77) after month 3. There was no significant between-group difference (*p* = 0.761). A mixed model analysis revealed significant correlation between the telangiectasia grade and the mean LMNA values at baseline, 1 week, 1 month, 2 months, and 3 months (*p*<*0.001*).

### Corneal Staining and Meibum Quality

In the injection group, corneal staining decreased significantly from 1.47 to 0.83 at 1 week post-treatment (mean change, −0.68, 95% CI −1.08 to −0.28, *p* = 0.001), and this change was maintained at 3 months post-treatment (*p* = 0.344) (Supplementary Table 1). In the eye drop group, corneal staining decreased significantly from 0.87 to 0.57 at 3 months (mean change, −0.38, 95% CI −0.69 to −0.06, *p* = 0.021). Improvements in corneal staining were significantly better in the injection group than in the eye drop group at 1 week, 1 month, and 2 months post-treatment (*p* <0.05); however, no between-group difference was observed at 3 months (*p* = 0.675).

In the injection group, the meibum quality score significantly improved from 19.02 to 16.21 at week 1 (mean change, −2.81, 95% CI −4.47 to −1.15, *p* = 0.001), and this improvement was maintained at 3 months (*p* = 0.127). In the eye drop group, the meibum quality score significantly improved from 18.79 to 15.67 at month 1 (mean change, −3.12, 95% CI −5.38 to −0.87, *p* = 0.007). This improvement was maintained at 3 months and was significantly better than that in the injection group (*p* = 0.021) (Supplementary Table 2).

### Meiboscore and Conjunctival Redness

The meiboscore significantly decreased from 2.21 to 2 at 1 week post-treatment in the injection group (mean change, −0.12, 95% CI −0.21 to −0.02, *p* = 0.017), and this improvement was maintained at 3 months post-treatment (*p* <0.001). In the eye drop group, the meiboscore significantly decreased from 1.68 to 1.62 at 1 month (mean change, −0.15, 95% CI −0.27 to −0.03, *p* = 0.012), which also persisted until 3 months post-treatment (*p* = 0.845). The decrease in meiboscore in the injection group was considerably greater than that in the eye drop group at 2 (*p* = 0.012) and 3 months (*p* <0.001) (Supplementary Table 2).

In the injection group, conjunctival redness values significantly decreased from 0.77 to 0.37 (mean change, −0.47, 95% CI −0.73 to −0.2, *p* = 0.001) at month 1, persisting until 3 months post-treatment (*p* = 0.089). However, the eye drop group exhibited a significant decrease in conjunctival redness at 3 months post-treatment (mean change, −0.32, 95% CI −0.58 to −0.06, *p* = 0.017). No between-group differences in conjunctival redness were observed at any of the post-treatment visits (Supplementary Table 2).

### Fluorescein Break-Up Time and Lipid Layer Thickness

In the injection group, FBUT increased significantly from 3.64 to 4.96 s at month 2 (mean change 0.96, 95% CI 0.11–1.8, *p* = 0.027) and remained stable at month 3 (*p* = 0.667) (Supplementary Table 2). However, FBUT remained at the baseline value at every visit for the eye drop group (*p* > 0.05). Despite this, the FBUT was significantly higher in the injection group than in the eye drop group at 1 month (mean difference 1.25, 95% CI 0.21–2.29, *p* = 0.019).

LLT did not significantly differ from baseline in either group at any visit, and the between-group difference was insignificant at month 3 (Supplementary Table 2).

### Safety and Compliance

No systemic AEs were detected in any patient. In the injection group, the most common AE at postoperative day 1 was dot hemorrhage at the injection site (16.7%). Among patients receiving eye drops, the most common AEs were eye irritation and transient eye redness, detected in 13.3 and 16.7% of patients at months 1 and 2, respectively. No local AEs were observed at month 3 in either group.

There was no significant between-group difference in the frequency of lid hygiene and the use of artificial tears at 3 months. At month 3, patients in the eye drop group received bevacizumab 3.78 times per day on average (Supplementary Table 3).

## Discussion

This is the first prospective, open-label, observer-blinded randomized controlled trial to investigate the ability of bevacizumab (eye drops or intra-MG injection) to improve lid margin telangiectasia and the signs and symptoms of MGD-associated posterior blepharitis. The results indicated that, when performed in conjunction with regular lid hygiene, a single intra-MG injection of 2.5% bevacizumab can significantly reduce telangiectasia grade and LMNA, and that bevacizumab eye drops can significantly decrease the telangiectasia grade within 3 months. Compared with the eye drop group, the injection group demonstrated improvements in corneal staining, meiboscore, and FBUT. However, both groups exhibited significant improvements in dry eye symptoms and clinical signs, including corneal staining, meibum quality, meiboscore, and conjunctival redness. Our results are consistent with those of previous studies (11, 18, 19).

In the injection group, telangiectasia grading decreased from 1 month to 3 months post-treatment. A similar trend was observed in the eye drop group beginning in month 2 following treatment initiation. Despite of this improvement, there was no difference in decreasing telangiectasia grade between groups at the end of study. Consistently with telangiectasis grade, significant changes in the LMNA values were observed in the injection group. Although our data did not reveal a significant change in LMNA in the eye drop group, we observed a significant correlation between the telangiectasia grade and LMNA in every follow-up time point. The telangiectasia grade is a widely accepted qualitative method for assessing clinical outcomes; however, a previous study (12) demonstrated that telangiectasia grading performed by general ophthalmologists had only moderate reliability. Therefore, quantitative, computer-assisted measurements of LMNA may represent a new strategy for diagnosis and post-treatment monitoring. However, this parameter requires further studies in order to determine the sensitivity and specificity. Further, differences in ethnicity or skin color may influence the ability to detect changes in blood vessels.

The OSDI scores decreased significantly from week 1 to month 3, although there were no between-group differences. Previous studies of bevacizumab therapy in DED and MGD (11, 18, 19) showed significantly improve dry eye symptoms. Improvements in OSDI scores may be explained by decreases in corneal staining and lid margin inflammation and by improvements in tear film stability.

Inflammation is among the numerous pathophysiological processes associated with MGD-associated posterior blepharitis. *In vitro*, stimulation of conjunctival epithelial and fibroblast cells by increasing levels of inflammatory cytokines in turn increases VEGF production (20). One human study (21) reported that patients with MGD exhibited significantly higher VEGF levels than did healthy volunteers. The higher VEGF levels would stimulate neovascularization (22); inflammation (23); and lymphangiogenesis (24), which might be related to the pathogenic mechanisms of DED (25–27). In addition, VEGF and VEGF receptor 2 are involved in the pathogenesis of neuropathic pain (28). Bevacizumab treatment can reduce dry eye symptoms within as little as 1 week and our study reported mean OSDI scores of <13 at week 1 and month 1 in the eye drop group. In comparison, the earliest improvements in dry eye symptoms are observed at week 2 in patients treated with 5% lifitegrast (29) and after 1 month (30) in patients treated with cyclosporine eye drops (CsA).

**Figure 5:**
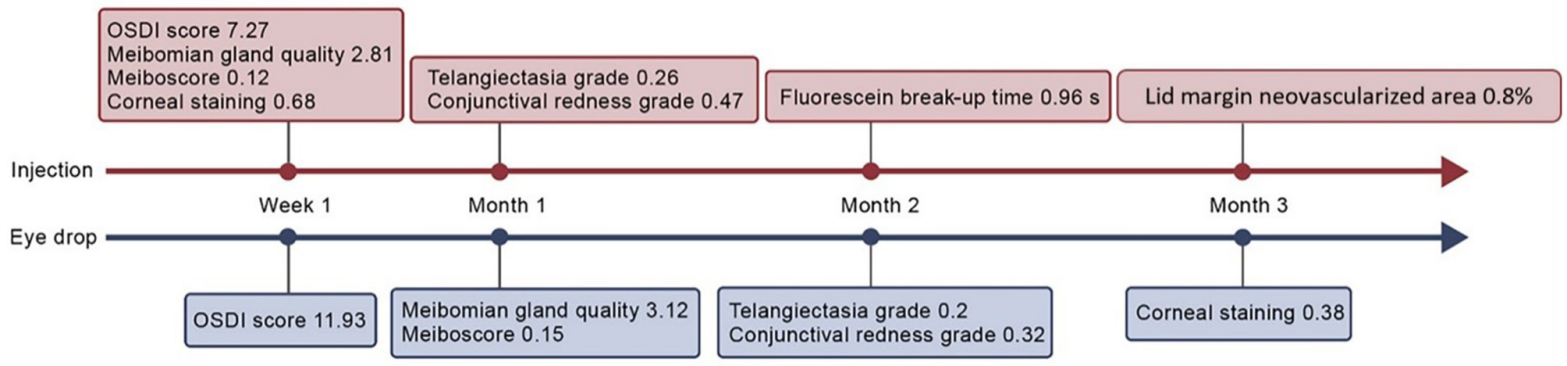
Anticipated clinically significant improvements for the eight outcomes.

In the injection group, meibum quality significantly improved to its maximum level at week 1 and gradually decreased until month 3. However, in the eye drop group, an increasingly significant improvement in meibum quality was observed from months 1 to 3, with better improvement than that in the injection group at month 3. This is in line with the meiboscore results. Previous studies (31, 32) observed that participants who underwent intense pulsed light (IPL) treatment exhibited significant reductions in levels of tear cytokines. These reductions were positively correlated with improvements in meibum quality, meibum expression, and meiboscore changes, consistent with findings in a previous study (33). Furthermore, Arita et al. (17). explained that the dark lesions observed in non-contact meibography may be attributable to degenerative meibum, aside from MG dropout. Together, these findings suggest that bevacizumab treatment can reduce inflammation, which may in turn improve meibum quality, meiboscores, and the integrity of the tear film lipid layer (31) (Figure 5).

Conjunctival redness began to decrease from month 1 in the injection group and month 2 in the eye drop group. This reduction was consistent with telangiectasia grading and can be explained by multiple anti-VEGF mechanisms. Changes in the size of blood vessels and areas of neovascularization may take longer to occur depending on the administration route, drug concentration, and extent of drug penetration. These factors may explain why bevacizumab eye drops are as effective as injected bevacizumab despite the slower initial responses of patients. However, eye drops are advantageous in that they are non-invasive and suitable for routine treatment.

In the injection group, corneal staining decreased significantly from week 1 to month 3; in the eye drop group, there was significant improvement at 3 months post-treatment. However, the duration over which this reduction was maintained was shorter than that reported for other anti-inflammatory medications. For instance, 5% lifitegrast can improve the inferior corneal staining score at month 3 (34), whereas CsA can reduce corneal staining from month 1 (35) to 4.

The LLT cut-off value (36) for screening obstructive MGD ( ≤ 75 nm) had a sensitivity of 65.8% and a specificity of 63.4%. Moreover, LLT is affected by age, sex, and other factors. In our study, the mean LLT of both groups was consistent with that reported in previous studies. Moreover, there was no significant change in LLT in either group at 3 months post-treatment (31). However, a longer monitoring period may be required to observe changes in LLT.

In our study, 0.05% bevacizumab eye drops caused ocular irritation and transient eye redness in 13.3 and 3.3% of patients, respectively. No local AEs were observed in either group at 3 months. In comparison, CsA can cause burning at the instillation site in up to 29% of patients (37), whereas lifitegrast has been reported to cause dysgeusia in 16.2% of patients (29).

Further randomized, placebo-controlled studies are required to determine the suitable concentration, frequency, and duration of treatment and to compare levels of inflammatory cytokines and nerve fiber density between baseline and various follow-up periods.

In conclusion, the present findings demonstrate that lid hygiene combined with an intra-MG injection of bevacizumab or bevacizumab eye drops is safe and effective for the treatment of lid margin telangiectasia, dry eye symptoms, and clinical signs of MGD-associated posterior blepharitis. We recommend intra-MG injections as a suitable treatment for patients with MGD-associated posterior blepharitis who exhibit moderate to severe lid margin telangiectasia or poor compliance with topical eye drops. In addition, LMNA and the anticipated clinically significant improvements in the eight outcomes addressed in this study may aid in monitoring the response to bevacizumab treatment.

## Data Availability Statement

The raw data supporting the conclusions of this article will be made available by the authors, without undue reservation.

## Ethics Statement

The studies involving human participants were reviewed and approved by King Chulalongkorn Memorial Hospital's Institutional Review Board (IRB Certificate of Approval No. 947/2020). The patients/participants provided their written informed consent to participate in this study.

## Author Contributions

CT: data curation, format analysis, funding acquisition, investigation, methodology, project administration, resources, validation, and writing original draft. NK: conceptualization, funding acquisition, methodology, supervision, validation, and review and editing. PC: data curation. KP: conceptualization, methodology, supervision, validation, and review and editing. All authors attest that they meet the current ICMJE criteria for authorship.

## Funding

This study was primarily supported by the Ratchadapiseksompotch Fund, Faculty of Medicine, Chulalongkorn University (grant number RA63/094). The funding body was not involved in conducting this study.

## Conflict of Interest

The authors declare that the research was conducted in the absence of any commercial or financial relationships that could be construed as a potential conflict of interest.

## Publisher's Note

All claims expressed in this article are solely those of the authors and do not necessarily represent those of their affiliated organizations, or those of the publisher, the editors and the reviewers. Any product that may be evaluated in this article, or claim that may be made by its manufacturer, is not guaranteed or endorsed by the publisher.

## References

[1] Lekhanont K Rojanaporn D Chuck RS Vongthongsri A. Prevalence of dry eye in Bangkok, Thailand. Cornea. (2006) 25:1162–7. 10.1097/01.ico.0000244875.92879.1a 17172891

[2] Lin PY Cheng CY Hsu WM Tsai SY Lin MW Liu JH. Association between symptoms and signs of dry eye among an elderly Chinese population in Taiwan: the Shihpai eye study. Invest Ophthalmol Visual Sci. (2005) 46:1593–8. 10.1167/iovs.04-0864 15851556

[3] Nelson JD Shimazaki J Benitez-del-Castillo JM Craig JP McCulley JP Den S. The international workshop on meibomian gland dysfunction: report of the definition and classification subcommittee. Invest Ophthalmol Visual Sci. (2011) 52:1930–7. 10.1167/iovs.10-6997b 21450914 PMC3072158

[4] Arita R Fukuoka S. Efficacy of azithromycin eyedrops for individuals with meibomian gland dysfunction-associated posterior blepharitis. Eye Contact Lens. (2021) 47:54–9. 10.1097/ICL.0000000000000729 32649390 PMC7752207

[5] Kyei S Asiedu K Ephraim RKD Adanusa M. Posterior blepharitis and associated potential factors: a study among pregnant women. Ocular Immunol Inflam. (2021) 2021:1–7. 10.1080/09273948.2021.1896007 33683981

[6] Geerling G Tauber J Baudouin C Goto E Matsumoto Y O'Brien T. The international workshop on meibomian gland dysfunction: report of the subcommittee on management and treatment of meibomian gland dysfunction. Invest Ophthalmol Visual Sci. (2011) 52:2050–64. 10.1167/iovs.10-6997g 21450919 PMC3072163

[7] Alghamdi YA Camp A Feuer W Karp CL Wellik S Galor A. Compliance and subjective patient responses to eyelid hygiene. Eye Contact Lens. (2017) 43:213–7. 10.1097/ICL.0000000000000258 27243349 PMC5124421

[8] Romero JM Biser SA Perry HD Levinson DH Doshi SJ Terraciano A. Conservative treatment of meibomian gland dysfunction. Eye Contact Lens. (2004) 30:14–9. 10.1097/01.ICL.0000095229.01957.89 14722463

[9] Koenig Y Bock F Horn F Kruse F Straub K Cursiefen C. Short- and long-term safety profile and efficacy of topical bevacizumab (Avastin) eye drops against corneal neovascularization. Graefe Arch Clin Exp Ophthalmol. (2009) 247:1375–82. 10.1007/s00417-009-1099-1 19415316

[10] Dastjerdi MH Al-Arfaj KM Nallasamy N Hamrah P Jurkunas UV Pineda R. Topical bevacizumab in the treatment of corneal neovascularization: results of a prospective, open-label, noncomparative study. Arch Ophthalmol. (2009) 127:381–9. 10.1001/archophthalmol.2009.18 19365012 PMC2703579

[11] Jiang X Wang Y Lv H Liu Y Zhang M Li X. Efficacy of intra-meibomian gland injection of the anti-VEGF agent bevacizumab for the treatment of meibomian gland dysfunction with lid-margin vascularity. Drug Design Dev Therapy. (2018) 12:1269–79. 10.2147/DDDT.S146556 29805249 PMC5960246

[12] Arita R Minoura I Morishige N Shirakawa R Fukuoka S Asai K. Development of definitive and reliable grading scales for meibomian gland dysfunction. Am J Ophthalmol. (2016) 169:125–37. 10.1016/j.ajo.2016.06.025 27345733

[13] Huang ST Tian BS Xiao O Yang YJ Zhou SY. Safety of antivascular endothelial growth factor administration in the ocular anterior segment in pterygium and neovascular glaucoma treatment: systematic review and meta-analysis. Medicine. (2018) 97:e11960. 10.1097/MD.0000000000011960 30142821 PMC6112962

[14] Schiffman RM Christianson MD Jacobsen G Hirsch JD Reis BL. Reliability and validity of the ocular surface disease index. Arch Ophthalmol. (2000) 118:615–21. 10.1001/archopht.118.5.615 10815152

[15] Murphy PJ Lau JS Sim MM Woods RL. How red is a white eye? Clinical grading of normal conjunctival hyperaemia. Eye. (2007) 21:633–8. 10.1038/sj.eye.6702295 16518366

[16] Bron AJ Evans VE Smith JA. Grading of corneal and conjunctival staining in the context of other dry eye tests. Cornea. (2003) 22:640–50. 10.1097/00003226-200310000-00008 14508260

[17] Arita R. Meibography: a Japanese perspective. Invest Ophthalmol Visual Sci. (2018) 59:Des48–55. 10.1167/iovs.17-23631 30481806

[18] Jiang X Lv H Qiu W Liu Z Li X Wang W. Efficiency and safety of subconjunctival injection of anti-VEGF agent - bevacizumab - in treating dry eye. Drug Design Dev Therapy. (2015) 9:3043–50. 10.2147/DDDT.S85529 26109847 PMC4472070

[19] Kasetsuwan N Chantaralawan K Reinprayoon U Uthaithammarat L. Efficacy of topical bevacizumab 0.05% eye drops in dry eye disease: a double-masked, randomized trial. PLoS ONE. (2020) 15:e0234186. 10.1371/journal.pone.0234186 32502179 PMC7274382

[20] Nagineni CN William A Cherukuri A Samuel W Hooks JJ Detrick B. Inflammatory cytokines regulate secretion of VEGF and chemokines by human conjunctival fibroblasts: role in dysfunctional tear syndrome. Cytokine. (2016) 78:16–9. 10.1016/j.cyto.2015.11.016 26615568 PMC12023846

[21] Enríquez-de-Salamanca A Castellanos E Stern ME Fernández I Carreño E García-Vázquez C. Tear cytokine and chemokine analysis and clinical correlations in evaporative-type dry eye disease. Mol Vision. (2010) 16:862–73. 20508732 PMC2874579

[22] Philipp W Speicher L Humpel C. Expression of vascular endothelial growth factor and its receptors in inflamed and vascularized human corneas. Invest Ophthalmol Visual Sci. (2000) 41:2514–22. 10937562

[23] Yoo SA Kwok SK Kim WU. Proinflammatory role of vascular endothelial growth factor in the pathogenesis of rheumatoid arthritis: prospects for therapeutic intervention. Med Inflam. (2008) 2008:129873. 10.1155/2008/129873 19223981 PMC2638142

[24] Cursiefen C Chen L Borges LP Jackson D Cao J Radziejewski C. VEGF-A stimulates lymphangiogenesis and hemangiogenesis in inflammatory neovascularization via macrophage recruitment. J Clin Invest. (2004) 113:1040–50. 10.1172/JCI20465 15057311 PMC379325

[25] Goyal S Chauhan SK El Annan J Nallasamy N Zhang Q Dana R. Evidence of corneal lymphangiogenesis in dry eye disease: a potential link to adaptive immunity? Arch Ophthalmol. (2010) 128:819–24. 10.1001/archophthalmol.2010.124 20625040 PMC3089983

[26] Stevenson W Chauhan SK Dana R. Dry eye disease: an immune-mediated ocular surface disorder. Arch Ophthalmol. (2012) 130:90–100. 10.1001/archophthalmol.2011.364 22232476 PMC3677724

[27] Goyal S Chauhan SK Dana R. Blockade of prolymphangiogenic vascular endothelial growth factor C in dry eye disease. Arch Ophthalmol. (2012) 130:84–9. 10.1001/archophthalmol.2011.266 21911653 PMC3629840

[28] Lin J Li G Den X Xu C Liu S Gao Y. VEGF and its receptor-2 involved in neuropathic pain transmission mediated by P2X_2_(/)3 receptor of primary sensory neurons. Brain Res Bulletin. (2010) 83:284–91. 10.1016/j.brainresbull.2010.08.002 20705122

[29] Tauber J Karpecki P Latkany R Luchs J Martel J Sall K. Lifitegrast ophthalmic solution 5.0% versus placebo for treatment of dry eye disease: results of the randomized phase III OPUS-2 study. Ophthalmology. (2015) 122:2423–31. 10.1016/j.ophtha.2015.08.001 26365210

[30] Sall K Stevenson OD Mundorf TK Reis BL. Two multicenter, randomized studies of the efficacy and safety of cyclosporine ophthalmic emulsion in moderate to severe dry eye disease. CsA phase 3 study Group. Ophthalmology. (2000) 107:631–9. 10.1016/S0161-6420(99)00176-1 10768324

[31] Choi M Han SJ Ji YW Choi YJ Jun I Alotaibi MH. Meibum expressibility improvement as a therapeutic target of intense pulsed light treatment in meibomian gland dysfunction and its association with tear inflammatory cytokines. Sci Rep. (2019) 9:7648. 10.1038/s41598-019-44000-0 31113979 PMC6529521

[32] Liu R Rong B Tu P Tang Y Song W Toyos R. Analysis of cytokine levels in tears and clinical correlations after intense pulsed light treating meibomian gland dysfunction. Am J Ophthalmol. (2017) 183:81–90. 10.1016/j.ajo.2017.08.021 28887117

[33] Ban Y Shimazaki-Den S Tsubota K Shimazaki J. Morphological evaluation of meibomian glands using noncontact infrared meibography. Ocular Surface. (2013) 11:47–53. 10.1016/j.jtos.2012.09.005 23321359

[34] Sheppard JD Torkildsen GL Lonsdale JD D'Ambrosio FA Jr McLaurin EB. Lifitegrast ophthalmic solution 5.0% for treatment of dry eye disease: results of the OPUS-1 phase 3 study. Ophthalmology. (2014) 121:475–83. 10.1016/j.ophtha.2013.09.015 24289915

[35] Chen M Gong L Sun X Xie H Zhang Y Zou L. A comparison of cyclosporine 0.05% ophthalmic emulsion versus vehicle in Chinese patients with moderate to severe dry eye disease: an eight-week, multicenter, randomized, double-blind, parallel-group trial. J Ocul Pharmacol Ther. (2010) 26:361–6. 10.1089/jop.2009.0145 20698799

[36] Finis D Pischel N Schrader S Geerling G. Evaluation of lipid layer thickness measurement of the tear film as a diagnostic tool for meibomian gland dysfunction. Cornea. (2013) 32:1549–53. 10.1097/ICO.0b013e3182a7f3e1 24097185

[37] Labetoulle M Leonardi A Amrane M Ismail D Garrigue JS Garhöfer G. Persistence of efficacy of 0.1% cyclosporin a cationic emulsion in subjects with severe keratitis due to dry eye disease: a nonrandomized, open-label extension of the SANSIKA study. Clin Therap. (2018) 40:1894–906. 10.1016/j.clinthera.2018.09.012 30389343

