# Supplementary material for: Bevacizumab Eye Drops Vs. Intra-meibomian Gland Injection of Bevacizumab for Meibomian Gland Dysfunction-Associated Posterior Blepharitis
Source: Front Med (Lausanne). 2022 Jun 10;9:895418. doi: 10.3389/fmed.2022.895418 (PMC9226372; doi:10.3389/fmed.2022.895418)
Supplement: Supplementary file 1 [file Table_1.pdf]

Supplementary Table 1. Ocular Surface Disease Index

OSDI (0–100)	Injection group			Eye drop group			Between treatment	
	Mean ± SD	Mean change (95% CI)	*p*-value	Mean ± SD	Mean change (95% CI)	*p*-value	Mean difference (95% CI)	*p*-value
Baseline	25.45 ± 14.28	Reference	1	23.73 ± 9.94	Reference	1	Reference	1
1 week	18.18 ± 11.13	-7.27 (-11.11, -3.42)	<0.001*	11.73 ± 8.15	-11.93 (-16.15, -7.7)	<0.001*	4.66 (-1.06, 10.38)	0.11
1 month	16.06 ± 10.64	-9.39 (-13.24, -5.55)	<0.001*	12.04 ± 7.36	-11.47 (-15.69, -7.24)	<0.001*	2.07 (-3.65, 7.79)	0.478
2 months	15.82 ± 10.38	-10.04 (-13.89, -6.19)	<0.001*	14.46 ± 11.28	-9.12 (-13.35, -4.9)	<0.001*	-0.92 (-6.64, 4.8)	0.753
3 months	17.15 ± 10.15	-8.5 (-12.43, -4.57)	<0.001*	14.02 ± 14.24	-9.47 (-13.7, -5.25)	<0.001*	0.99 (-4.79, 6.77)	0.738

Patient-reported outcome

OSDI, Ocular Surface Disease Index, *p<0.05
