# Supplementary material for: Bevacizumab Eye Drops Vs. Intra-meibomian Gland Injection of Bevacizumab for Meibomian Gland Dysfunction-Associated Posterior Blepharitis
Source: Front Med (Lausanne). 2022 Jun 10;9:895418. doi: 10.3389/fmed.2022.895418 (PMC9226372; doi:10.3389/fmed.2022.895418)
Supplement: Supplementary file 2 [file Table_2.pdf]

Supplementary Table 2. Secondary Outcomes

Variables	Injection group			Eye drop group			Between treatment	
	Mean ± SD	Mean change (95% CI)	*p*-value	Mean ± SD	Mean change (95% CI)	*p*-value	Mean difference (95% CI)	*p*-value
**Corneal staining (0–5)**								
Baseline	1.47 ± 1.27	Reference	1	0.87 ± 1.09	Reference	1	Reference	1
1 week	0.83 ± 0.86	-0.68 (-1.08, -0.28)	0.001*	0.83 ± 0.65	-0.17 (-0.49, 0.15)	0.306	-0.52 (-1.03, -0.01)	0.048*
1 month	0.93 ± 1.16	-0.63 (-1.03, -0.23)	0.002*	0.77 ± 0.78	0.02 (-0.3, 0.34)	0.918	-0.65 (-1.16, -0.14)	0.013*
2 months	0.57 ± 0.65	-0.83 (-1.23, -0.43)	<0.001*	0.97 ± 0.85	-0.05 (-0.37, 0.27)	0.759	-0.78 (-1.29, -0.27)	0.003*
3 months	1.14 ± 1.39	-0.49 (-0.89, -0.08)	0.02*	0.57 ± 0.59	-0.38 (-0.69, -0.06)	0.021*	-0.11 (-0.63, 0.41)	0.675
**Meibum quality (0–24)**								
Baseline	19.02 ± 3.82	Reference	1	18.79 ± 3.74	Reference	1	Reference	1
1 week	16.21 ± 2.51	-2.81 (-4.47, -1.15)	0.001*	17.93 ± 3.58	-0.86 (-3.12, 1.4)	0.456	-1.95 (-4.75, 0.85)	0.173
1 month	16.68 ± 4.95	-2.34 (-4, -0.68)	0.006*	15.67 ± 6.61	-3.12 (-5.38, -0.87)	0.007*	0.79 (-2.02, 3.59)	0.583
2 months	16.93 ± 3.82	-2.09 (-3.75, -0.43)	0.014*	15.9 ± 5.72	-2.89 (-5.15, -0.64)	0.012*	0.81 (-2, 3.61)	0.573
3 months	17.43 ± 3.18	-1.49 (-3.18, 0.2)	0.084	13.95 ± 4.64	-4.84 (-7.09, -2.58)	<0.001*	3.34 (0.51, 6.17)	0.021*

Variables	Injection group			Eye drop group			Between treatment	
	Mean ± SD	Mean change (95% CI)	*p*-value	Mean ± SD	Mean change (95% CI)	*p*-value	Mean difference (95% CI)	*p*-value
**Meiboscore (0–6)**								
Baseline	2.21 ± 1.42	Reference	1	1.68 ± 1.11	Reference	1	Reference	1
1 week	2 ± 1.31	-0.12 (-0.21, -0.02)	0.017*	1.63 ± 1.15	-0.05 (-0.17, 0.07)	0.404	-0.07 (-0.22, 0.09)	0.404
1 month	1.87 ± 1.25	-0.24 (-0.33, -0.15)	<0.001*	1.62 ± 1.16	-0.15 (-0.27, -0.03)	0.012*	-0.09 (-0.24, 0.06)	0.254
2 months	1.75 ± 1.24	-0.35 (-0.45, -0.26)	<0.001*	1.56 ± 1.17	-0.15 (-0.27, -0.04)	0.01*	-0.2 (-0.35, -0.04)	0.012*
3 months	1.59 ± 1.28	-0.5 (-0.6, -0.41)	<0.001*	1.57 ± 1.18	-0.14 (-0.26, -0.02)	0.021*	-0.37 (-0.52, -0.21)	<0.001*
**Conjunctival redness (0–4)**								
Baseline	0.77 ± 0.78	Reference	1	0.73 ± 0.7	Reference	1	Reference	1
1 week	0.6 ± 0.63	-0.22 (-0.48, 0.05)	0.107	0.57 ± 0.42	-0.17 (-0.43, 0.09)	0.208	-0.05 (-0.42, 0.32)	0.791
1 month	0.37 ± 0.58	-0.47 (-0.73, -0.2)	0.001*	0.63 ± 0.69	-0.1 (-0.36, 0.16)	0.45	-0.37 (-0.74, 0)	0.052
2 months	0.6 ± 0.81	-0.3 (-0.56, -0.04)	0.026*	0.5 ± 0.82	-0.23 (-0.49, 0.03)	0.078	-0.07 (-0.44, 0.3)	0.724
3 months	0.39 ± 0.66	-0.49 (-0.75, -0.22)	<0.001*	0.4 ± 0.71	-0.32 (-0.58, -0.06)	0.017*	-0.17 (-0.54, 0.2)	0.375

Variables	Injection group			Eye drop group			Between treatment	
	Mean ± SD	Mean change (95% CI)	*p*-value	Mean ± SD	Mean change (95% CI)	*p*-value	Mean difference (95%CI)	p-value
**FBUT (s)**								
Baseline	3.64 ± 1.52	Reference	1	4.88 ± 1.64	Reference	1	Reference	1
1 week	4.8 ± 1.57	0.66 (-0.18, 1.51)	0.124	4.82 ± 2.01	0.05 (-0.56, 0.66)	0.873	0.62 (-0.43, 1.66)	0.247
1 month	4.67 ± 2.04	0.81 (-0.04, 1.66)	0.061	4.31 ± 0.93	-0.44 (-1.05, 0.17)	0.156	1.25 (0.21, 2.29)	0.019*
2 months	4.96 ± 1.91	0.96 (0.11, 1.8)	0.027*	4.64 ± 1.39	0.01 (-0.6, 0.62)	0.974	0.95 (-0.1, 1.99)	0.075
3 months	5.12 ± 2.21	0.77 (-0.1, 1.63)	0.082	4.29 ± 1.97	-0.11 (-0.72, 0.5)	0.73	0.89 (-0.16, 1.94)	0.098
**LLT (nm)**								
Baseline	64 ± 26.24	Reference	1	72.33 ± 27.17	Reference	1	Reference	1
1 week	62.33 ± 31.38	-2.63 (-14.16, 8.89)	0.654	69.67 ± 27.24	0.43 (-9.69, 10.55)	0.933	-3.07 (-18.4, 12.27)	0.695
1 month	60.6 ± 30.96	-4.07 (-15.59, 7.46)	0.489	59.27 ± 26.66	-3.4 (-13.52, 6.72)	0.51	-0.67 (-16, 14.67)	0.932
2 months	56.07 ± 34.66	-4.4 (-15.93, 7.13)	0.454	65.33 ± 27.49	-0.07 (-10.19, 10.05)	0.99	-4.33 (-19.67, 11)	0.58
3 months	56.14 ± 32.9	-8.51 (-20.28, 3.25)	0.156	56.67 ± 27.92	-9.13 (-19.25, 0.99)	0.077	0.64 (-14.86, 16.13)	0.936

Clinician subjective and objective outcomes

FBUT, fluorescein break-up time; s, second; LLT, lipid layer thickness; nm, nanometer, *p<0.05
