# Supplementary material for: Bevacizumab Eye Drops Vs. Intra-meibomian Gland Injection of Bevacizumab for Meibomian Gland Dysfunction-Associated Posterior Blepharitis
Source: Front Med (Lausanne). 2022 Jun 10;9:895418. doi: 10.3389/fmed.2022.895418 (PMC9226372; doi:10.3389/fmed.2022.895418)
Supplement: Supplementary file 3 [file Table_3.pdf]

Supplementary Table 3. Compliance for Lid Hygiene Care and Use of Tear Substitutes

Variables	Injection group (n=15)	Eye drop group (n=15)	*p*-value
**Lid hygiene care (day/week)**			
1 week	4.53 ± 3.04	6.33 ± 1.19	0.042*
1 month	5.93 ± 1.83	6.2 ± 1.32	0.651
2 months	6.43 ± 0.86	5.3 ± 1.93	0.047*
3 months	6.29 ± 0.91	5.37 ± 1.91	0.115
**Use of tear substitutes (drop/day)**			
1 week	4.33 ± 2.41	3.23 ± 2.65	0.244
1 month	3.27 ± 2.46	2.37 ± 2	0.281
2 months	3.67 ± 2.01	2.07 ± 1.88	0.033*
3 months	3.61 ± 2.11	2.57 ± 1.84	0.168

*$p<0.05$
